# Dysplastic Megakaryocytes in a Gallbladder Neck Lymph Node in a Patient With Essential Thrombocythemia: A Case Report

**DOI:** 10.1155/crh/5591025

**Published:** 2026-03-05

**Authors:** Mahi Balcı

**Affiliations:** ^1^ Department of Pathology, Faculty of Medicine, Kırıkkale University, Kırıkkale, Turkey, kku.edu.tr

**Keywords:** dysplastic megakaryocytes, essential thrombocythemia, extramedullary hematopoiesis, gallbladder, lymph node

## Abstract

A woman in her 60s with a known history of JAK2‐positive essential thrombocythemia presented with a one‐week history of jaundice. Laboratory testing revealed elevated bilirubin and alkaline phosphatase levels. Abdominal ultrasonography identified a 1‐cm gallstone, and laparoscopic cholecystectomy was performed. Histopathological evaluation revealed chronic cholecystitis along with a 5‐mm lymph node in the gallbladder neck harboring atypical megakaryocytes with multinucleation and nuclear lobulation. Immunohistochemistry confirmed megakaryocytic origin with strong positivity for CD61 and supportive positivity for Factor VIII, while epithelial and lymphoid markers were negative, ruling out metastatic carcinoma or lymphoma. This case underscores the rare involvement of lymph nodes by dysplastic megakaryocytes in essential thrombocythemia and highlights the importance of considering hematologic malignancies in the differential diagnosis of lymphadenopathy.

## 1. Introduction

Essential thrombocythemia (ET) is a myeloproliferative neoplasm (MPN) frequently associated with mutations in the Janus kinase 2 (JAK2) gene and shares molecular and clinical features with polycythemia vera, primary myelofibrosis, and other MPNs [1]. Although extramedullary hematopoiesis (EMH) is relatively uncommon in ET, mobilization of neoplastic or non‐neoplastic hematopoietic stem and progenitor cells into the peripheral circulation may facilitate hematopoietic activity in extramedullary sites such as the spleen, liver, and lymph nodes [2].

Rarely, EMH may occur in atypical anatomical locations, creating diagnostic challenges because it can closely mimic metastatic carcinoma or lymphoma. We report a rare case of dysplastic megakaryocytic involvement of a gallbladder neck lymph node in a patient with JAK2‐positive ET. Notably, no other hematopoietic lineages were identified within the lymph node, emphasizing the importance of clinicopathological correlation and appropriate immunohistochemical evaluation in establishing the correct diagnosis.

## 2. Case Presentation

A female patient in her 60s presented to the general surgery clinic with a one‐week history of jaundice. Laboratory tests revealed elevated total bilirubin (1.21 mg/dL), direct bilirubin (0.4 mg/dL), indirect bilirubin (0.8 mg/dL), and alkaline phosphatase (119 U/L). Peripheral blood values showed a white blood cell count of 10.15 × 10^3^/μL, a red blood cell count of 12 × 10^6^/μL, and a platelet count of 399 × 10^3^/μL. Abdominal ultrasonography identified a single 1‐cm gallstone, and the patient subsequently underwent laparoscopic cholecystectomy.

The patient had a known history of JAK2‐positive ET. At the time of initial presentation and preoperative evaluation, no splenomegaly or hepatomegaly was documented. Splenomegaly was reported during subsequent postoperative clinical follow‐up. The postoperative course was uneventful, with no reported complications.

The resected gallbladder measured 7 × 7.3 cm with a wall thickness of 0.2 cm. The specimen contained an 8‐mm gallstone and a 5‐mm lymph node located in the gallbladder neck region. Histopathological examination of the gallbladder revealed features consistent with chronic cholecystitis.

**FIGURE 1:**
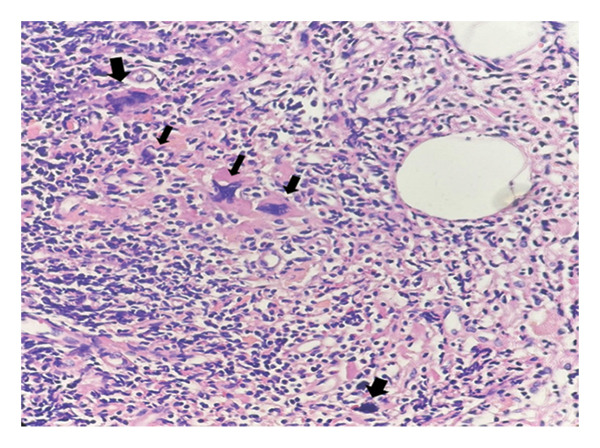
Clusters of atypical megakaryocytes with abundant eosinophilic cytoplasm, irregular nuclear contours, and hyperchromatic smudged nuclei (arrows) embedded in fibrotic stroma (H&E, × 200; scale bar: 50 μm).

**FIGURE 2:**
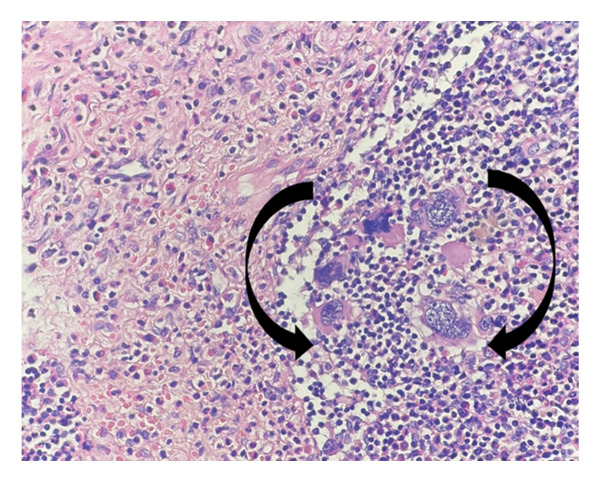
Megakaryocytes with multilobulated and hyperchromatic nuclei, some showing apoptotic features, located within lymphoid parenchyma (arrows) (H&E, × 400; scale bar: 20 μm).

**FIGURE 3:**
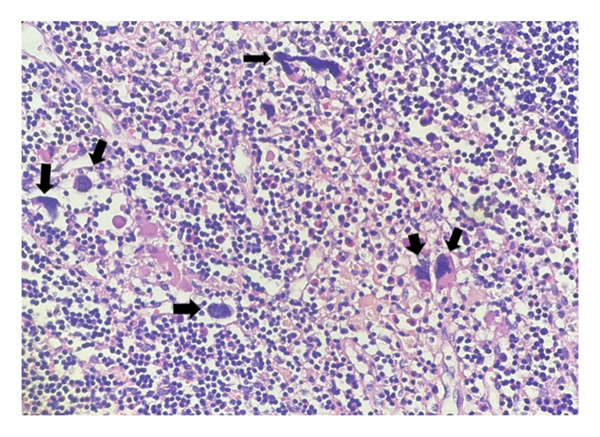
Numerous dysplastic megakaryocytes exhibiting apoptotic‐like morphology and nuclear irregularities dispersed throughout the lymphoid tissue (arrows) (H&E, × 200; scale bar: 50 μm).

Histological evaluation of the lymph node demonstrated clusters of atypical cells with multinucleation and nuclear lobulation within fibrotic and hemorrhagic stroma, raising suspicion for either epithelial or megakaryocytic origin (Figures 1, 2, and 3). The overall lymph node architecture was preserved. Dysplastic megakaryocytes were present focally, comprising approximately 10%–15% of the nodal tissue. No accompanying erythroid or myeloid hematopoietic precursors were identified.

**FIGURE 4:**
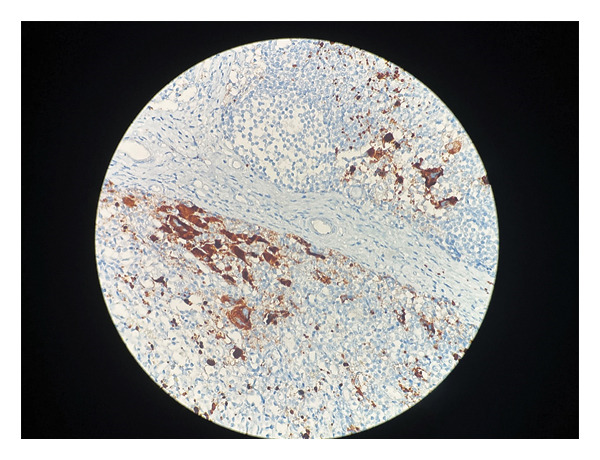
Immunohistochemical staining for CD61 demonstrating strong cytoplasmic positivity in dysplastic megakaryocytes (IHC, × 200; scale bar: 50 μm).

**FIGURE 5:**
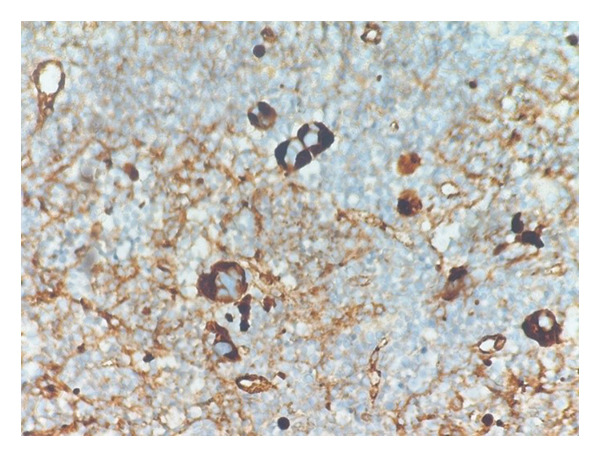
Immunohistochemical staining for Factor VIII–related antigen highlighting cytoplasmic positivity in megakaryocytic cells with accompanying capillary endothelial reactivity (IHC, × 400; scale bar: 20 μm).

Immunohistochemical analysis demonstrated strong cytoplasmic positivity for CD61 (GPIIIa), confirming megakaryocytic lineage (Figure 4). Factor VIII–related antigen was also positive and served as a supportive marker (Figure 5). The atypical cells were negative for cytokeratin, CD34, CD45, CD3, CK7, CD20, CD68, S100, and CD15, effectively excluding epithelial malignancies and other hematologic disorders. Review of the clinical history confirmed a diagnosis of JAK2‐positive ET made 10 years earlier. Collectively, these findings were consistent with lymph node involvement by ET as part of a MPN, reflecting migration of dysplastic megakaryocytes rather than metastatic carcinoma.

## 3. Discussion

Lymphadenopathy in patients with ET can present diagnostic challenges due to the morphological overlap between megakaryocytic proliferation and malignant conditions such as metastatic carcinoma, sarcoma, or lymphomas such as Hodgkin lymphoma. In this case, atypical megakaryocytic clusters in a gallbladder neck lymph node raised suspicion for metastatic disease. However, immunohistochemical markers played a critical role in distinguishing diagnoses.

Factor VIII–related antigen has long been recognized as a useful immunohistochemical marker in the identification of megakaryocytes and has played an important role in hematopathology practice. Its expression may also be observed in endothelial cells, and its sensitivity can vary depending on the tissue context. Therefore, interpretation of Factor VIII staining benefits from correlation with morphology and the use of additional lineage‐specific markers.

In contemporary hematopathology practice, as reflected in the WHO 5th Edition and the International Consensus Classification (ICC 2022), CD61 (GPIIIa) is widely regarded as a highly sensitive marker for confirming megakaryocytic lineage, particularly in extramedullary sites [3]. CD42b (GPIbα) is often recommended as a complementary marker to enhance specificity. In the present case, strong CD61 positivity provided clear confirmation of megakaryocytic differentiation, while Factor VIII–related antigen served as a supportive immunohistochemical marker in conjunction with the morphological findings.

When considering metastatic carcinoma, especially pancreaticobiliary or gastrointestinal tumors, cytokeratins (CK7 and CK20) are key markers of epithelial malignancies. The absence of cytokeratin expression in megakaryocytes ruled out a carcinoma metastasis. Similarly, CD15 and CD30 are classical markers for Reed–Sternberg cells in Hodgkin lymphoma and were negative here, supporting megakaryocytic origin.

Megakaryocytes in lymph nodes may occur in both MPN and non‐MPN settings, representing a spectrum of EMH. In MPNs such as ET, this reflects aberrant clonal expansion driven by mutations in genes such as *JAK2*, *CALR*, or *MPL* [4]. Hematopoietic stem and progenitor cell trafficking, regulated by sphingosine‐1‐phosphate receptor 1 (S1P1) and the chemokine receptor CXCR4 through interaction with its ligand CXCL12, is central to this process [5]. Dysregulation of these pathways, together with permissive inflammatory microenvironments, facilitates pathological hematopoiesis at extramedullary sites.

Nonhepatosplenic EMH (NHS‐EMH) occurs in atypical organs such as the central nervous system, ovaries, skin, lungs, pleura, pericardium, and lymph nodes [2, 6, 7]. These rare presentations underscore the influence of both intrinsic molecular alterations and extrinsic inflammatory factors. Chronic inflammation, such as cholecystitis in the present case, may create a permissive microenvironment that supports dysplastic megakaryocytic proliferation.

The dysplastic features observed, including multinucleation, nuclear hyperlobulation, and architectural disorganization, indicate a neoplastic process rather than a purely reactive one. Traditionally, the term “extramedullary hematopoiesis” describes hematopoietic activity occurring outside the bone marrow; however, this broad definition may fail to fully encompass the pathological clonal proliferation seen in MPNs such as ET. Moreover, there is conceptual ambiguity whether the presence of atypical megakaryocytes in extramedullary sites represents an abnormal migration of dysplastic precursor cells (a form of pathological “precursor cell trafficking”) or direct mobilization of dysplastic megakaryocytes themselves. Therefore, the term “extramedullary dysmegakaryocytic proliferation” might more accurately reflect the underlying pathological process by emphasizing the clonal, neoplastic nature of the megakaryocytic expansion outside the marrow.

From a clinical management perspective, identification of dysplastic megakaryocytes within a lymph node is particularly relevant for oncologists and pathologists, as this finding does not represent metastatic disease, lymphomatous involvement, or tumor‐related nodal staging. Misinterpretation may lead to inappropriate upstaging, unnecessary investigations, or unwarranted oncologic treatment; therefore, accurate recognition is essential to avoid diagnostic and therapeutic errors.

Current evidence does not support extramedullary or circulating megakaryocytic involvement as an independent adverse prognostic factor in ET. To date, no association has been demonstrated with differences in overall survival, risk of fibrotic progression, leukemic transformation, or thrombotic events. Rather than indicating disease progression, the presence of dysplastic megakaryocytes in extramedullary sites is best interpreted as a manifestation of the systemic and heterogeneous nature of ET. While such findings may coexist with other extramedullary features during the disease course, including splenomegaly, a direct predictive or causal relationship cannot be established. Consequently, careful clinicopathologic correlation and longitudinal follow‐up are warranted, without implying adverse prognostic significance.

## 4. Conclusion

This case highlights a rare instance of isolated dysplastic megakaryocytic involvement in a gallbladder neck lymph node in a patient with ET. Recognition of this finding is clinically important, as it may mimic metastatic carcinoma or lymphoma and lead to inappropriate staging or treatment if misinterpreted.

## Author Contributions

The author was solely responsible for the conception, data collection, analysis, and manuscript preparation.

## Funding

The author has nothing to report.

## Ethics Statement

The author has nothing to report.

## Consent

Written informed consent was obtained from the patient for publication of this case report and accompanying images.

## Conflicts of Interest

The author declares no conflicts of interest.
